# Mortality of neonates born to mothers of extreme reproductive age in Ethiopia; multilevel mixed effect analysis of Ethiopian demographic and health survey data of 2016

**DOI:** 10.3389/fped.2024.1390952

**Published:** 2024-06-28

**Authors:** Berhan Tekeba, Masresha Asmare Techane, Belayneh Shetie Workneh, Alebachew Ferede Zegeye, Almaz Tefera Gonete, Tewodros Getaneh Alemu, Mulugeta Wassie, Alemneh Tadesse Kassie, Mohammed Seid Ali, Enyew Getaneh Mekonen, Tadesse Tarik Tamir

**Affiliations:** ^1^ Department of Pediatrics and Child Health Nursing, School of Nursing, College of Medicine and Health Sciences, University of Gondar, Gondar, Ethiopia; ^2^ Department of Emergency and Critical Care Nursing, School of Nursing, College of Medicine and Health Sciences, University of Gondar, Gondar, Ethiopia; ^3^ Department of Medical Nursing, School of Nursing, College of Medicine and Health Sciences, University of Gondar, Gondar, Ethiopia; ^4^ School of Nursing, College of Medicine and Health Sciences, University of Gondar, Gondar, Ethiopia; ^5^ Department of Clinical Midwifery, School of Midwifery, College of Medicine and Health Sciences, University of Gondar, Gondar, Ethiopia; ^6^ Department of Surgical Nursing, School of Nursing, College of Medicine and Health Sciences, University of Gondar, Gondar, Ethiopia

**Keywords:** neonatal mortality, extreme reproductive age, multilevel mixed effect analysis, demographic and health survey, Ethiopia

## Abstract

**Introduction:**

Neonatal mortality is still a major public health problem in middle- and low-income countries like Ethiopia. Despite strategies and efforts made to reduce neonatal death, the mortality rate declines at a slower pace in the country. Though there are studies conducted on neonatal mortality and its determinants, our searches of the literature have found no study on the extent of mortality of neonates born to mothers of extreme reproductive age in the study area. Therefore, this study aimed to assess the magnitude and factors associated with the mortality of neonates born to mothers of extreme reproductive age in Ethiopia.

**Methods:**

Secondary data analysis was conducted using 2016 Ethiopian Demographic and Health Survey data. The final study contained an overall weighted sample of 2,269 live births. To determine the significant factors in newborn deaths, a multilevel binary logistic regression was fitted. For measuring the clustering impact, the intra-cluster correlation coefficient, median odds ratio, proportional change in variance, and deviation were employed for model comparison. The adjusted odds ratio with a 95% confidence interval was presented in the multivariable multilevel logistic regression analysis to identify statistically significant factors in neonatal mortality. A *P*-value of less than 0.05 was declared statistically significant.

**Results:**

The neonatal mortality rate of babies born to extreme aged reproductive women in Ethiopia was 34 (95% Cl, 22.2%–42.23%) per 1,000 live birth. Being twin pregnancy (AOR = 10; 95% Cl: 8.61–20.21), being from pastoralist region (AOR = 3.9; 95% Cl: 1.71–8.09), having larger baby size (AOR = 2.93; 95% Cl: 1.4–9.12) increase the odds of neonatal mortality. On the other hand, individual level media exposure (AOR = 0.3; 95% Cl: 0.09–0.91) and community level media exposure (AOR = 0.24; 95% Cl: 0.07–0.83), being term gestation (AOR = 0.14; 95% Cl: 0.01–0.81) decreases the odds of neonatal mortality born to mothers of extreme reproductive age.

**Conclusion:**

Ethiopia had a greater rate of neonatal death among babies born at the extremes of reproductive age than overall reproductive life. Multiple pregnancies, larger baby sizes, emerging regions, term gestation, and media exposure were found to be significant factors associated with the mortality of neonates born to mothers of extreme reproductive age. Therefore, the concerned bodies should give emphasis to mothers giving birth before the age of 20 and above 35, access to media, healthy pregnancy, and special attention to pastoralists to reduce the burden of neonatal mortality.

## Introduction

Neonatal mortality is defined as the death of newborn with in the first 28 days of life. Extreme maternal age also refers to maternal age of less than twenty (teenagers) and advanced maternal age (35 and older) (1). Extreme maternal age are associated with a higher risk of neonatal mortality, infant and under five mortality (2–4). The neonatal period is the most vulnerable time for the child's survival (5). The global neonatal mortality decreased by 52% from 36.6 deaths per 1,000 live birth in 1900, to 17.5 in 2019 (2). However, still it is estimated that 6.3 million children death globally occur during the first 28 days of life and almost all (98%) of these occurs in developing countries (6).

For the last few decades, important progress has been made to save the children in the world. Due to this, the rate of neonatal death declined by 54%, from 37 in 1990 to 17 per 1,000 live births in 2020 (7). However, the rate declines at a slower pace in Ethiopia (8, 9), particularly marginally higher in pastoralist/emerging regions (10, 11). The most recent Ethiopian Demographic and Health Survey indicate that the rate of neonatal death decreased from 49 per 1,000 live births to 29 per 1,000 live births. This is less than the rate of decline for infant and under-five mortality, which was 97 to 48 and 166 to 67, respectively (10, 12, 13).

According to different studies conducted, obstetric, socio-demographic, reproductive health, medical, behavioral, and nutritional-related factors are common factors associated with neonatal mortality (14). In addition there exists a relationship between maternal health and neonatal survival. For instance, poor maternal nutritional and health status has been linked to poor neonatal outcome and this is influenced by determinants such as socio-economic, demographic and biologic factors (15).

The Ethiopian government strives to meet Sustainable Development Goal (SDG)-3, which requires all nations to eradicate preventable newborn deaths, with as few as 12 per 1,000 live births being the threshold. Additionally, the nation launched and is currently implementing the Health Sector Transformation Plan, which aims to lower newborn mortality to 10 per 1,000 live births by the program's end (16). Nevertheless, the problem still persists and the declination lag is very slow (17).

Globally, about 25% of women give birth to their first child before turning 20; in developing countries, this percentage is even higher. Early sexual initiation, low self-esteem, and/or isolation from social endeavors can all lead to teenage pregnancy. Unwanted pregnancies were linked to increased rates of school dropout, unemployment, psychological risk, pregnancy termination, and disdain for parental care in this age range. Contrarily, pregnancy in women over 35 is a prominent trend in this era for a number of reasons, such as women's evolving social roles in the workforce and within the home, their desire of financial security, and their accomplishment of a high level of education. Social, economic, and emotional maturity lead to a deeper understanding of the importance of effective pregnancy monitoring. Nevertheless, they became a risk factor for late pregnancy (1, 18). Both young and older maternal ages are associated with poor maternal and newborn outcomes. Thus, the degree and depth of maternal age influence on neonatal health should be understood in order to address the issue and increase neonatal survival.

According to earlier studies, extremes in a mother's age are among the socio-demographic characteristics of mothers that are known to be associated with poor delivery outcomes (19). Extreme maternal age has been linked to poor maternal and perinatal outcomes. As an illustration, being an older mother is linked to negative outcomes for both the mother and the child, including a higher chance of maternal diabetes and hypertension, congenital abnormalities, pregnancy complications, premature birth, and cesarean delivery (20). Conversely, younger women were linked to adverse socioeconomic circumstances, preterm, perinatal mortality, intra-uterine growth restriction (IUGR), low birth weight, and major congenital anomalies (21).

Nevertheless, despite research on neonatal death and its determinants having been conducted in various regions of Ethiopia, the mortality of neonates born to mothers of extreme reproductive age was not well studied. In addition, to the best of the investigator's search of information, the predictors of neonatal mortality born to mothers of extreme reproductive age have not been determined yet. Thus, this study aimed to assess the magnitude and contributing factors of neonatal mortality born to mothers of extreme reproductive age in Ethiopia, which helps responsible bodies make appropriate and timely interventions.

## Methods

### Study design, period and setting

A secondary analysis of data from the 2016 Ethiopian Demographic and Health Survey was used to conduct a population-based cross-sectional study. The data were collected from nine regional states and two city administrations (Addis Ababa and Dire Dawa). The region was subdivided into 68 zones, 817 districts, and 16,253 Kebele (the lowest administrative units in the country), and the study was conducted from January 18 to June 27, 2016. The 2016 Ethiopian Demographic and Health Survey was a cross-sectional survey that examined population health with a focus on maternal and child health, as well as population health indicators of global interest. The Ministry of Health and Ethiopia's Central Statistical Agency (CSA) worked together to collect the data. A stratified, two-stage cluster sampling technique was applied. In the first stage, 645 enumeration areas (202 urban and 443 rural EAs) were selected, with probability sampling proportional to the size of the EAs. Secondly, fixed numbers of 28 households per cluster were randomly selected. A total of 18,008 households were randomly selected, and 15,683 eligible women were interviewed. Data on 2,269 live births were extracted from Ethiopian Demographic and Health Survey (EDHS) in 2016. The full EDHS 2016 report included information on the sampling process as well as the general data collection (10).

### Sample and populations

The source population was all live births among extreme-aged reproductive women (<20 and above 35 years old) within 28 days of life in Ethiopia, whereas all live babies aged 0–28 days in the enumeration areas (EAs) were the study populations. A two-stage stratified cluster sampling technique was employed. Stratification was done by separating each region into urban and rural areas. In stage one, 645 EAs were selected using probability sampling. In the second stage, households were selected systematically in each enumeration area. Then, newborns were selected using the Kids Record (KR) file, and important variables were selected from the data set.

### Study variables

The outcome variable of this study was neonatal mortality. Neonatal mortality is defined as the death of a neonate within twenty-eight days of birth, which was dichotomized as yes = 1 for neonates who had died within twenty-eight days of birth, whereas no = 0 for neonates who were alive within one month of life. The neonatal mortality was calculated as the number of infant death that occurs between 0 and 27 days of life divided by the number of life birth, multiplied by 1,000 (22). As reviewed from different literature, extreme maternal age is considered young for mothers less than 20 years old, and older maternal age is considered as maternal age greater than 35 years old (1).

### Independent variables

Both individual and community-level factors were reviewed from different literatures, and these include types of pregnancy, maternal age, BMI and educational level, place and mode of delivery, types of gestation, ANC visits, media exposure, and household wealth quintiles. Region, place of residence, community ANC utilization, community women's education, distance to health facilities, community media exposure, and community wealth status were community-level factors aggregated from individual-level factors (23). ANC was classified as optimal if a mother had more than 4 visits during her pregnancy time (24). Maternal education status was categorized as no formal education, primary, secondary and above in our study (25). Region were classified as pastoralist dominant, agrarian dominant and city dwellers (26). Reading newspapers, watching television, and listening to the radio were three ways that media exposure was computed. When there was exposure to any of the three, these variables were combined and classified as yes, meaning that reading newspapers, listening to the radio, or watching television were present. Distance to health facilities was categorized as “yes” for big problems to access and “no” for women to access health facilities without difficulty. Birth size was categorized as small for a weight less than 2,500 g, normal for a weight of 2,500–3,900 g, and large for a weight greater than 4,000 g. Maternal body max index (BMI) was found from DHS recorded data and determined during the survey period as the ratio of weight in kilogram to the square of height in meter (kg/m^2^) and available in the data as two digit decimal then we categorized the recorded BMI as low for BMI < 18.49 kg/m^2^, normal for 18.5–24.9 kg/m^2^, and large for ≥25 kg/m^2^ (27).

### Data collection procedure

The DHS Program granted us permission to collect and use the data from http://www.dhsprogram.com for this study after we asked permission. Before releasing the data collected from the DHS to the public, participant identification was erased, and institutional ethical approval was waived to ensure compliance with the rules governing the protection of human beings.

### Data management and model selection

Following extraction from the EDHS portal, data were entered, coded, cleaned, recorded, and analyzed using Stata version 14. In DHS, data variables are nested by clusters, and those within the same cluster show more similarities than those with separate clusters. Consequently, the assumption of independent observation and equal variance across clusters violates the conventional logistic regression model. Hence, there is a need for a more advanced model to account for cluster factors. A model called multi-level multivariable logistic regression was employed to identify factors associated with neonatal mortality. Accordingly, multi-level mixed effect logistic regression uses four models: the null model, model I (individual level factors), model II (community level factors), and model III (which holds both individual and community level factors). The model without an exposure variable (the null model) was used to check the variability of neonatal mortality across the cluster. The associations of individual-level factors with the outcome variable (model II) were examined. In model III, or the final model, the association of both individual and community-level variables was fitted with the outcome variable. Random effects, or measures of variation of the outcome variable, were estimated by the median odds ratio (MOR), intra-class correlation coefficient (ICC), and proportional change in variance (PCV). The intra-class correlation coefficient (ICC) and proportional change in variance (PCV) were computed to measure the variation between clusters. Taking clusters as a random variable, the ICC reveals the variation of early neonatal death between clusters as: ICC = V_C_/(V_C _+ 3.29) × 100%, where V_C_ is the variance of the cluster. The MOR is the median value of the odds ratio between the area of highest risk and the area of lowest risk for neonatal death when two clusters are randomly selected, using clusters as a random variable; MOR = e^95√Vc^. Moreover, the PCV demonstrates the variation in the prevalence of neonatal death as explained by factors and computed as PCV = (V_null_−V_C_)/V_null_ × 100%, where V_null_ is the variance of the null model and V_C_ is the cluster-level variance. The fixed effects were used to estimate the association between the likelihood of neonatal death and individual and community-level independent variables. It was assessed, and the strength was presented using an adjusted odds ratio (AOR) and 95% confidence intervals with a *p*-value of 0.05. Because of the nested nature of the model, deviation = −2 (log likelihood ratio) was used to compare models, and the model with the lowest deviance was selected as the best-fit model. The variables used in the models were verified for multi-collinearity by measuring the variance inflation factors (VIF), with the findings falling within acceptable limits of one to ten (28–30).

## Result

### Socio-demographic characteristics

A total of 2,269 mothers with extreme maternal age (less than twenty and above thirty-five) were included in the study. The majority (82.19%) of mothers were aged above 35 years (older mothers), and nearly one fifth (17.81%) of mothers were aged below 20 years old (teenagers). Nearly two-thirds (72.45%) of mothers hadn't received formal education. More than half of the participants’ partners or husbands (58.39%) lacked formal education. The majority of participants (86%) reside in rural areas. Majorities (97%) of children's were delivered by normal vaginal delivery and were born at home (71.22%). The majority of newborns (98.5%) were born at nine months (term) Table 1.

**Table 1:** Socio-demographic, obstetric, child and maternal characteristics.

Variables	Response	Frequency	%
Maternal age	<20 years	404	17.81
	≥35 years	1,865	82.19
Maternal education	No education	1,644	72.45
	Primary	484	21.33
	Secondary and above	141	6.21
Paternal education	No education	1,221	58.39
	Primary	600	28.69
	Secondary and above	270	12.91
Child sex	Male	1,160	51.12
	Female	1,109	48.88
Place of delivery	Home	1,616	71.22
	Health-facility	653	28.78
Place of residence	Rural	316	13.93
	Urban	1,953	86.07
House hold head sex	Male	1,744	76.86
	Female	525	23.14
Wealth index	Poor	1,272	56.06
	Middle	365	16.09
	Rich	632	27.85
No-of under-five in household	None	94	4.14
	One	1,025	45.17
	Two	827	36.45
	Three and above	323	14.24
Types of pregnancy	Single	2,189	96.47
	Multiple	80	3.53
Breast feed status	Non breast feed	1,356	59.76
	Breastfeed	913	40.25
Pregnancy complication	Yes	459	45.00
	No	561	55.00
History of terminated pregnancy	Yes	244	89.25
	No	2,025	10.75
PNC check	Yes	146	7.67
	No	1,693	88.97
Birth order	First	370	16.31
	Second/third	189	8.33
	Fourth and above	1,710	75.36
ANC visit	Optimal	1,098	51.61
	Non optimal	1,171	48.39
Pregnancy wanted	Yes	1,996	87.97
	No	273	12.03
Maternal BMI	<18.49 kg/m^2^	557	24.55
	18.5–24.9 kg/m^2^	1,406	61.97
	≥25 kg/m^2^	306	13.49
Media exposure	Yes	659	29.04
	No	1,610	70.96
Community level factors			
Place of residence	Rural	1,953	86.07
	Urban	316	13.93
Distance to health facility	Big problem	1,263	55.66
	Not big problem	1,006	44.34
Region	Agrarian	1,285	56.63
	Pastoralists	819	36.10
	Urbanism	165	7.27
Community ANC	Low	1,122	49.45
	High	1,147	50.55
Community illiteracy	Low	882	38.87
	High	1,387	61.13
Community poverty	Low	1,217	46.36
	High	1,052	53.64
Community media exposure	High	1,355	59.72
	Low	914	40.28

### Prevalence of neonatal mortality among extreme reproductive aged women’s in Ethiopia

According to this study, neonatal mortality among extreme-aged mothers in Ethiopia was 34 (95% Cl, 27.2–42.2) per 1,000 live births. This newborn mortality rate is higher than Ethiopia's general reproductive age women's neonatal death rate, which is 29 per 1,000 live births and 30 per 1,000 live births, according to the 2016 and 2019 EDHS reports, respectively (31).

### Random effect and model comparison

Random effect or community variation was examined by ICC, MOR, and PCV. The ICC value in model I was 0.8, indicating that 80% of neonatal mortality among the extremes of reproductive age in Ethiopia was attributable to the differences at the cluster level. The median odds ratio in the null model was 2.33, which indicated that neonatal mortality was different between clusters. The PCV in the final model indicates that 56.25% of neonatal mortality was attributable to both individual and community-level factors. Finally, the four models were compared to select the best fit model, and the final model with the lowest deviance was considered the best fit model, which is model IV (149.52) (Table 2).

**Table 2:** Random effect and model fit statistics of neonatal mortality among extreme reproductive age in Ethiopia.

Parameter	Model I	Model II	Model III	Model IV
Variance	0.80	0.98	0.71	0.35
ICC	19.55%	22.95%	17.76%	9.6%
MOR	2.33	2.56	2.23	1.75
PCV	Reference	21.72%	9.5%	56.25%
LLR	−333.54	−82.83	−329.28	−74.76
Deviance	667.08	165.65	658.56	149.52

ICC, Intra-cluster coefficient; MOR, median odds ratio; LLR, log likelihood ratio.

### Factors associated with neonatal mortality among extreme age of reproductive age

Socio-demographic, obstetric, and child-related factors were analyzed in the bivariate multiple logistic regression model. The variables associated with neonatal mortality among women of extreme reproductive age at a *p*-value of 0.2 were further analyzed in multi-variable multilevel mixed effect models (model III and model IV). In the final model (model IV), both individual and community-level factors were fitted to control confounders and identify statistically significant factors of neonatal mortality among women of extreme reproductive age.

Accordingly, the final model revealed that mothers having term pregnancy had 86% (AOR: 0.14, 95% CI, 0.01–0.81) lower odds of neonatal mortality than mothers having non-term pregnancy. Similarly, women's having media exposure was 70% (AOR: 0.3, 95% CI: 0.09–0.91) less likely to have neonatal mortality than women's not exposed to media. On the other hand, children with a large birth size had almost three times (AOR: 2.93, 95% CI, 1.4–9.12) higher odds of neonatal mortality than normal birth-sized babies’. Mothers having multiple gestations had a tenfold (AOR: 10.5% CI, 8.61–20.21) more likely neonatal death than singleton gestation. Among community-level factors, Ethiopian pastoralist regions were almost fourfold (AOR: 3.94, 95% CI: 1.71–8.09) more likely to have neonatal mortality than mothers who reside in the city. On the other hand, communities’ having media exposure had 76% (AOR: 0.24 95% CI, 0.07–0.83) less likely neonatal morality than communities with no media exposure (Table 3).

**Table 3:** Multivariable multi-level logistic regression analysis of individual and community level factors associated with neonatal mortality among extreme reproductive age women in Ethiopia, 2016.

Individual and community level factors		Model II AOR (95% Cl)	Model III AOR (95% Cl)	Model IV AOR (95% Cl)
Household head sex	Male	2.79 (0.41–19.12)		1.85 (0.29–11.86)
	Female	1		1
Place of delivery	Home	0.60 (0.20–1.86)		0.5 (0.16–1.53)
	Health facility	1		1
Child sex	Male	2.10 (0.70–6.33)		2.13 (0.73–6.20)
	Female	1		1
Maternal education	Non educated	0.47 (0.04–5.25)		0.48 (0.04–6.28)
	Primary	0.26 (0.02–2.76)		0.18 (0.02–1.95)
	Secondary and above	1		1
Paternal education	Un-educated	7.90 (0.77–81.2)		5.92 (0.58–9.89)
	Primary	11.46 (1.27–10.3)		6.85 (0.76–61.6)
	Secondary and above	1		1
Duration of pregnancy	<9 month	1		1
	≥9 month	0.14 (0.02–1.14)		0.14 (0.01–0.81)*
Mode of delivery	Vaginal delivery	0.22 (0.04–1.31)		0.16 (0.02–1.09
	Cesarean section	1		1
Wanted pregnancy	Wanted	1		1
	Unwanted	1.33 (0.30–5.90)		2.24 (1.14–5.41)
ANC visit	Optimal	1.12 (0.38–3.36)		1.65 (0.50–5.41)
	Un-optimal	1		1
Maternal BMI	<18.49 kg/m^2^	1.27 (0.35–4.55)		1.13 (0.37–4.59)
	18.5–24.9 kg/m^2^	1		1
	≥25 kg/m^2^	1.05 (0.25–4.43)		1.23 (0.28–5.46)
Media exposure	Yes	2.52 (0.75–8.5)		0.3 (0.09–0.91)*
	No	1		1
Termination of PX history	Yes	2.41 (0.74–7.81)		2.49 (0.74–8.39)
	No	1		1
Pregnancy complication	Yes	0.47 (0.15–1.41)		0.49 (0.16–1.50)
	No	1		1
PNC check	Yes	0.36 (0.04–3.68)		0.27 (0.02–3.30)
	No	1		1
Birth size	Small	0.16 (0.02–1.6)		0.13 (0.01–1.24)
	Normal	1		1
	Large	3.9 (1.24–12.25)		2.93 (1.4–9.12)*
Wealth index	Rich	1		1
	Poor	0.63 (0.182.29)		0.38 (0.08–1.88)
	Middle	0.40 (0.08–2.10)		0.28 (0.05–1.39)
Birth order	First	1.63 (0.32–8.25)		1.81 (0.37–8.82)
	Second	2.9 (0.58–14.99)		3.38 (0.73–15.6)
	Third and above	1		1
Type of gestation	Single	1		1
	Multiple	8.4 (7.14–14.81)		10 (8.61–20.21)*
Community level factors				
Place of residence	Urban		1	1
	Rural		1.58 (0.57–4.35)	2.7 (0.34–22.67)
Distance to health facility	Big problem		0.81 (0.28–2.34)	0.63 (0.22–1.79)
	Not big problem		1	1
Community ANC Use	Low		0.92 (0.54–1.57)	1.84 (0.52–6.56)
	High		1	1
Community wealth status	Low		1	1
	High		0.96 (0.52–1.79**)**	0.65 (0.15–2.81)
Community illiteracy	Low		1	1
	High		0.92 (0.52–1.62)	2.01 (0.57–7.06)
Region	City dwellers		1	1
	Agrarians		4.37 (0.52–36.5)	1.9 (0.59–5.19)
	Pastoralists		3.14 (1.73–51.3)	3.94 (1.71–8.09)
Community media exposure	High		0.73 (0.41–1.3)	0.24 (0.07–0.83)
	Low		1	1

*Statistically significant (*p*-value < 0.05).

## Discussion

This study was done on newborns in Ethiopia whose moms were at the upper end of the reproductive age range; less than twenty and older than thirty-five. Thus, the study found that the neonatal mortality rates among extremes of reproductive age in Ethiopia were 34 (95% Cl, 27.2–42.2) per 1,000 live birth. This newborn mortality rate is higher than Ethiopia's general reproductive age women's neonatal mortality, which is 29 per 1,000 live births and 30 per 1,000 live births, according to the 2016 and 2019 EDHS reports, respectively (31). This is due to the fact that young mothers are more biologically vulnerable to unfavorable birth outcomes, such as physical immaturity causing pregnancy complications, and competition between the mother and the fetus for nutrients can exacerbate the effects of chronic malnutrition, leading to poorer outcomes for the health of the infant and child (32–35). On the other hand, older mothers are at a higher risk of fetal death at term from intra-partum asphyxia (36), more likely to experience unfavorable labor and delivery outcomes, obstetric difficulties, and preexisting medical conditions (37), including preterm delivery, perinatal mortality, low birth weight and macro-somia (38, 39). Therefore, older women and adolescent mothers deserve special consideration.

This study also found factors significantly associated with the mortality of neonates born to mothers of extreme age. Accordingly, media exposure, multiple gestations, larger birth size, duration of pregnancy, community media exposure, and region were significantly associated with neonatal death among the extremes of maternal and reproductive life.

The odds of neonatal death were higher among multiple births compared with singleton births. This is supported by study done in Korea (40). One possible explanation is that preterm births and low birth weight are commonly associated with twin pregnancies, which is the primary cause of neonatal mortality. Additionally, twin pregnancies expose the fetus to infection and twin-twin transfusion syndrome, which reduces infant survival and eventually causes death (35, 41, 42). It is consistent with findings in Ghana (43). This could be because preterm delivery and fetal growth restriction are associated with a higher likelihood of twin births, which in turn raises the risk of hypothermia, sepsis, and hypoglycemia, all of which may increase the risk of neonatal mortality (44). Thus, clinicians shall give special attention to multiple births through early detection of plurality by ultrasonography, give special care during delivery, and provide counseling and support to mothers on handling twin pregnancies during ANC and PNC visits.

Compared to full-term deliveries, non-term pregnancies experienced greater increases in neonatal, postnatal, and infant death rates (45, 46). An investigation conducted in New York supports the notion that preterm neonates were significantly more likely than full-term infants to experience hypoglycemia, respiratory distress, transient tachypnea, NICU need, respiratory intubation, intravenous fluid requirements, mechanical ventilation, and cesarean delivery, all of which could lead to serious complications or even death (47, 48). Thus, government and policymakers should pay attention to preterm births. Intensive care unit should be available for all preterm births.

According to this study, media exposure was significantly associated with neonatal mortality. Babies from parents with high media exposure at the individual and at the community level decreased the odds of neonatal death compared to newborns with low media exposure. This is in line with the study done in Bangladesh (49). One plausible explanation could be that moms and the general community, who were exposed to the media, had greater knowledge about the use of ANC, institutional delivery, and pediatric illnesses (50). Thus, the government and other relevant authorities should promote media exposure and increasing accesses to media.

Baby size at birth was also significantly associated with neonatal mortality. This is supported by study done in London (51) by which pregnancy with macrosomia increases the risk of maternal and neonatal complications, such as the necessity for an emergency cesarean section, obstetric brachial plexus injury, and birth fractures that compromise the newborn's quality of life (51). Furthermore, larger newborns are linked to other maternal co-morbidities including gestational diabetes, which puts the mother's and the child's lives in danger (52). Therefore, special consideration should be given to macrocosmic babies.

Regions in Ethiopia also significantly associated with neonatal mortality. Ethiopian regions can be classified as Agrarian, Pastoralist, and city administration.This is in line with the pervious study in Ethiopia (53). A plausible rationale could be because earlier research conducted in Ethiopia was conducted in pastoralist areas, where the availability of mother and child health services is relatively low and the population is economically disadvantaged compared to other regions (24). Moreover, these regions exhibit poorer vaccination rates, limited healthcare accessibility, lower urbanization, and higher incidence of infectious diseases such as acute respiratory infections and malaria in comparison to other regions of the country (54, 55). Hence, priority shall be given to pastoralists in increasing access to information regarding maternal and newborn care and in increasing access to basic health services to minimize the burden of neonatal mortality in these emerging regions of Ethiopia.

This study found that the newborn mortality rate is higher in extreme-aged reproductive women as compared to general reproductive-age women's neonatal deaths. The study also found that for babies born at extreme maternal age, factors at the individual and community levels were associated with neonatal mortality. Thus, responsible bodies shall give special attention to extremely aged reproductive women in Ethiopia and its predictors.

## Conclusion

The study found that for babies born at extreme maternal age, factors at the individual and community levels were associated with neonatal mortality. The main factors that determined neonatal death in our study were multiple pregnancies, larger babies, pastoral region, term gestation, and media exposure. Policy makers, researchers, health planners, and implementers must therefore place attention on promoting healthy pregnancy, promoting media access, and paying special attention to mothers in low and high quartiles of age.

### Strength and limitation

This study's strength was the use of multi-level modeling to make meaningful inferences and findings while accounting for the clustering effect in EDHS. The study has the potential to assist programmers and policymakers in developing effective national interventions because it is based on data from a countrywide survey. Due to the DHS nature of the data, variables like infectious illness, congenital abnormalities, and respiratory state after deliveries, which are thought to be the most common causes of neonatal mortality, were left out of the analysis. Furthermore, recall bias may exist in this study due to the cross-sectional nature of DHS and its reliance on respondents’ self-reports. Due to our small sample size of teenage mothers and our relatively larger sample of older mothers, which makes our sample inconsistent, we couldn't perform comparisons independently among these age groups; thus, a future comparative study on neonatal mortality between extreme maternal age and normal maternal age or between early (teenager maternal age) and older (advanced maternal age) shall be done.

## Data Availability

The datasets presented in this study can be found in online repositories. The data can be found here: https://www.dhsprogram.com/data/available-datasets.cfm.
